# The scoring bias in reverse docking and the score normalization strategy to improve success rate of target fishing

**DOI:** 10.1371/journal.pone.0171433

**Published:** 2017-02-14

**Authors:** Qiyao Luo, Liang Zhao, Jianxing Hu, Hongwei Jin, Zhenming Liu, Liangren Zhang

**Affiliations:** State Key Laboratory of Natural and Biomimetic Drugs, School of Pharmaceutical Sciences, Peking University, Beijing, P. R. China; National Chiao Tung University College of Biological Science and Technology, TAIWAN

## Abstract

Target fishing often relies on the use of reverse docking to identify potential target proteins of ligands from protein database. The limitation of reverse docking is the accuracy of current scoring funtions used to distinguish true target from non-target proteins. Many contemporary scoring functions are designed for the virtual screening of small molecules without special optimization for reverse docking, which would be easily influenced by the properties of protein pockets, resulting in scoring bias to the proteins with certain properties. This bias would cause lots of false positives in reverse docking, interferring the identification of true targets. In this paper, we have conducted a large-scale reverse docking (5000 molecules to 100 proteins) to study the scoring bias in reverse docking by DOCK, Glide, and AutoDock Vina. And we found that there were actually some frequency hits, namely interference proteins in all three docking procedures. After analyzing the differences of pocket properties between these interference proteins and the others, we speculated that the interference proteins have larger contact area (related to the size and shape of protein pockets) with ligands (for all three docking programs) or higher hydrophobicity (for Glide), which could be the causes of scoring bias. Then we applied the score normalization method to eliminate this scoring bias, which was effective to make docking score more balanced between different proteins in the reverse docking of benchmark dataset. Later, the Astex Diver Set was utilized to validate the effect of score normalization on actual cases of reverse docking, showing that the accuracy of target prediction significantly increased by 21.5% in the reverse docking by Glide after score normalization, though there was no obvious change in the reverse docking by DOCK and AutoDock Vina. Our results demonstrate the effectiveness of score normalization to eliminate the scoring bias and improve the accuracy of target prediction in reverse docking. Moreover, the properties of protein pockets causing scoring bias to certain proteins we found here can provide the theory basis to further optimize the scoring functions of docking programs for future research.

## Introduction

Small molecule drugs are rarely selective enough to interact solely with their designated targets. Known drugs have, on average, six molecular targets on which they exhibit activity[1, 2], usually resulting in unexpected side effects or toxicity[3–6]. On the other hand, the ability of small molecules to interact with multiple proteins also provides the basis to develop multitarget drugs[7, 8]. Thus, it is a critical step to identify all target proteins of small molecule drugs in drug discovery. Protein target prediction, also known as target fishing, helps to identify the potential targets of a query molecule. It may reveal targets of drugs with so far unknown mechanisms-of-action[9], contribute to rationally designing of less toxic or multitarget drugs[10–13], and reveal hidden opportunities in drug repurposing projects[14–16].

Target fishing includes experimental and computational approaches[17, 18]. Since the experimental target fishing is expensive and time-consuming, it is hard to predict all the possible targets from such a broad range of proteins in a short time. With the development of computing resources, computational target fishing has drawn more and more attentions in recent years[19]. Generally, computational target fishing can be classified as ligand-based methods and target-based methods[20]. Ligand-based methods simplify the problem to a similarity searching problem, and only use ligand information to predict target [21–23]. Target-based methods use the information of target proteins, which includes reverse docking, similarity comparison of protein sequences or binding pockets, and so on [24, 25]. Compared with other methods, reverse docking utilizes 3D protein structures and active site information to predict the binding mode as well as the binding energy of a ligand, which is a major advantage. With the rapid growth of the number of available 3D structures of proteins, reverse docking is becoming increasingly important in target fishing [26–30]. This approach has been demonstrated to be useful in target identification and some of the predicted results have been verified by bioassays and crystallographic studies. For example, a reverse docking study by Chen et al. used INVDOCK to identify therapeutic targets of medicinal herbal ingredients as well as synthetic chemicals and the majority of identified therapeutic targets have been confirmed[31]. In 2012, Eric et al. used Tarfisdock to conduct reverse docking against a pool of protein targets and identified the potential targets to rationalize the cytotoxic effects of aryl-aminopyridines and their derivatives[30]. These successful cases show that reverse docking has been playing an important role in protein target predictions of small molecules.

Although significant successes have been made in reverse docking, there are still some practical limitations for this method, such as the choice of an appropriate scoring function and the high false positive rate[32]. Especially, the scoring functions of current docking programs are designed for the virtual screening of small molecules, few of which is specifically optimized for reverse docking. As a result, the scoring functions would have scoring bias to some proteins with extreme properties, which accounts for the incomparability of docking scores for different proteins[33, 34]. In recent years, several attempts have been made to improve the accuracy of docking scores in reverse docking [32, 33, 35]. For example, Kellenberger et al. incorporated the topological molecular interaction fingerprint (IFP) into GOLD fitness score, improving the recall rate of true targets[35]. An evaluation of Glide in reverse docking on the Astex Diverse Set by Wang et al. showed the “interprotein scoring noise” of Glide scoring function and used a correction term containing the protein property “balance” to improve the accuracy rate of target predictions[33]. Thus, the docking score or the scoring function of current docking programs should be rationalized to suit the reverse docking for target fishing.

In this study, we conducted the reverse docking of 5000 small molecules to 100 proteins of DUD-E[36] using three common docking programs and found that there were some highly scored frequency hits in 100 proteins resulting from the scoring bias. After analyzing the binding pockets of these proteins, we found that they mostly had large protein-ligand contact area or high hydrophobicity. Then we proposed a score normalization strategy to eliminate this scoring bias in reverse docking. The score normalization strategy was validated with the Astex Diverse Set[37] containing 85 diverse protein-ligand complexes. The accuracy rate of reverse docking by Glide[38] was significantly improved after score normalization, demonstrating that the score normalization strategy is an effective approach to improve the success rate of target fishing. However, the accuracy rate of reverse docking by DOCK[39] and AutodockVina[40] had no obvious increase, because there might be other scoring defects in these two docking programs besides scoring bias. The research here would help to promote the application and development of reverse docking in target fishing.

## Materials and methods

### Dataset preparations

#### Standard protein dataset

Directory of useful decoys-enhanced (DUD-E)[36] is a benchmarking set that includes diverse targets such as GPCRs and ion channels, totaling 102 proteins with 22886 clustered decoys drawn from ChEMBL, each with 50 property-matched decoys drawn from ZINC. DUD-E is usually used to evaluate retrospective performance of classic docking-based virtual screening. Here, 100 proteins from DUD-E were chosen as standard protein dataset to study reverse docking, removing two highly homologous isoforms. The native ligands and water molecules of crystal protein structures were removed. Then all the proteins were prepared by the Protein Preparation module of Pipeline Pilot 7.5[41], which included adding hydrogens, protonation and repairing the missing or wrong residues.

#### Benchmark dataset

Available chemicals directory (ACD)[42] contains more than 8, 000, 000 compounds collected from more than 800 vendors, which covers almost all chemical space of exist compounds. We selected 5000 molecules randomly from ACD as benchmark dataset, which has the similar distribution of heavy atom number with ACD (S1 Fig in the Supporting Information). Thus, the benchmark dataset contains most molecular sizes of exist compounds, representing reverse docking cases of different molecular sizes. The benchmark dataset was prepared by the Ligprep[43] module of Schrödinger software, including adding hydrogens, ionization, generating isomers and energy minimization.

### Astex Diverse Set

Astex Diverse Set[37] is a diverse and high-quality test set containing 85 diverse and relevant protein-ligand complexes for the validation of protein-ligand docking performance. Here, Astex Diverse Set was selected as the validation dataset of docking score correction. The native ligands were extracted from crystal complexes and prepared by the Ligprep module of Schrödinger software like benchmark dataset. And 85 proteins were prepared by Protein Preparation module of Pipline Pilot 7.5 like standard protein dataset.

### Scoring functions of docking programs

The three docking programs (DOCK, Glide and AutoDock Vina) we chose here implement three different scoring functions, which could be used to study and compare their scoring ability in reverse docking. Since these three docking programs are popular in the field of molecular design, we aim to study the scoring performance in reverse docking by these three docking programs.

#### Grid score

The grid-based energy scoring function in DOCK is used as the docking score of receptors and ligands. The energy scoring component of DOCK is a type of force field scoring[39]. Force field scores are approximate molecular mechanics interaction energies, consisting of van der Waals and electrostatic components:

$$E=\sum_{i=1}^{lig}\sum_{j=1}^{rec}\left(\frac{A_{ij}}{r_{ij}^{a}}-\frac{B_{ij}}{r_{ij}^{b}}+332\frac{q_{i}q_{j}}{{Dr}_{ij}}\right),$$ (1)

Where each term is a double sum over ligand atoms *i* and receptor atoms *j*, *A*_*ij*_ and *B*_*ij*_ are van der Waals repulsion and attraction parameters, *r*_*ij*_ is the distance between atoms *i* and *j*, *q*_*i*_ and *q*_*j*_ are the point charges on atoms *i* and *j*, *D* is the dielectric function, and 332 is a factor that converts the electrostatic energy into kilocalories per mole.

#### Glide score

Glide score SP is used as the docking score of receptors and ligands by Glide, which is extended by the empirically based ChemScore function[38]. The scoring function of Glide score SP is as follows:

$$\Delta G_{bind}=C_{lipo-lipo}\sum f\left(r_{lr}\right)+C_{hbond-neut-neut}\sum g\left(\Delta \text{r}\right)h\left(\Delta \alpha \right)+C_{hbond-neut-charged}\sum g\left(\Delta \text{r}\right)h\left(\Delta \alpha \right)+C_{hbond-charged-charged}\sum g\left(\Delta \text{r}\right)h\left(\Delta \alpha \right)+C_{max-metal-ion}\sum f\left(r_{lm}\right)+C_{rotb}H_{rotb}+C_{polar-phob}V_{polar-phob}+C_{coul}E_{coul}+C_{vdW}E_{vdW}+solvation\;terms,$$ (2)

The lipophilic-lipophilic term is defined as in ChemScore, which extends over all ligand-atom/receptor-atom pairs. In Eq 2, *f*, *g*, and *h* are functions that give a full score (1.00) for distances or angles that lie within nominal limits and a partial score (1.00–0.00) for distances or angles that lie outside those limits but inside larger threshold values. The hydrogen-bonding term also uses the ChemScore form but is separated into differently weighted components that depend on whether the donor and acceptor are both neutral, one is neutral and the other is charged, or both are charged. The fifth term is metal-ligand interaction term, and the seventh term rewards instances in which a polar but non-hydrogen-bonding atom is found in a hydrophobic region. The second major component of Eq 2 is the incorporation of contributions from the Coulomb and vdW interaction energies between the ligand and the receptor. And the last term of Eq 2 is the introduction of a solvation model. Compared with other scoring functions, GlideScore SP combines the empirical-based and force-field-based scoring function to make the score more accuracy.

#### AutoDock Vina score

The general functional form of the conformation-dependent part of the scoring function AutoDock Vina is designed to work with is

$$c=\sum_{i<j}f_{t_{i}t_{j}}(r_{ij}),$$ (3)

where the summation is over all of the pairs of atoms that can move relative to each other, normally including 1–4 interactions, i.e., atoms separated by three consecutive covalent bonds. Here, each atom *i* is assigned a type *t*_*i*_, and a symmetric set of interaction function $f_{t_{i}t_{j}}$ of the interatomic distance *r*_*ij*_ should be defined. The derivation of AutoDock Vina scoring function combines certain advantages of knowledge-based potentials and empirical scoring funcitons: it extracts empirical information from both the conformational preferences of the receptor-ligand complexes and the experimental affinity measurements[40].

### Reverse docking procedure

The benchmark dataset and Astex Diverse Set defined 5000 and 85 reverse docking cases, respectively. In each case of benchmark dataset, one ligand was docked to 100 proteins of standard protein dataset. In each case of Astex Diverse Set, one ligand was docked to 85 proteins of Astex Diverse Set.

#### DOCK

All proteins of standard protein dataset and Astex Diverse Set were prepared by Dockprep module of Chimera 1.9[44] with default parameters before DOCK docking. The Sphgen module of DOCK 6.6[39] was used to generate the molecular surface of proteins and the spheres surrounding the proteins. The binding sites were selected based on the native X-ray ligands and the boxes with size of 10 Å × 10 Å ×10 Å at the binding sites were created. Preparation of the docking grids was then performed. Maximum iterations and maximum conformations were both set as 100. The docking calculation was performed and the top-10 scored conformations of each ligand were reserved. The best scored conformation was selected as the binding conformation of the ligand.

#### Glide

All the proteins of standard protein dataset and Astex Diverse Set were prepared by the Protein Preparation Wizard module of Schrödinger with OPLS_2005 force field. The receptor grid was generated for each protein as follows. The binding site of each protein was defined based on the native X-ray ligand and the enclosing box with the size limit of 8 Å was created. Other parameters used in the receptor grid generation were set to their defaults. Finally, ligands were docked to proteins using the “standard precision” mode of Glide[38]. For each ligand-protein pair, Glide reported 10 best binding conformations, and the conformation with lowest binding energy was selected.

#### AutoDock Vina

All the proteins of standard protein dataset and Astex Diverse Set as well as all the ligands were prepared by MGL Tools. The native X-ray ligand was set as the center of the enclosing box with the size of 22.5 Å × 22.5 Å × 22.5 Å. Docking calculations were performed using the default parameters implemented in AutoDock Vina 1.1.2[40]. Similarly, each ligand output 10 best binding conformations, and the conformation with lowest binding energy was selected.

### Protein pocket properties calculation

The properties of each protein binding pocket were calculated by the Sitemap[45] module of Schrödinger, including size, volume, exposure, enclosure, contact, phobic (representing hydrophobicity), philic (representing hydrophilicity), balance (representing the ratio of hydrophobicity and hydrophilicity) and don/acc (representing the ratio of hydrogen-bond donor and acceptor). In the research of our group, we have found that the contact area of protein-ligand complex is correlated with docking score. The contact area is defined as follows:

$$S_{contact}=\left(S_{protein}+S_{ligand}-S_{complex}\right)/2,$$ (4)

*S*_*contact*_, *S*_*protein*_, *S*_*ligand*_, and *S*_*complex*_ represent the surface area of contact, protein, ligand and complex, respectively.

Thus, contact area was calculated by an in-house python script and the median contact area of each protein with its all ligands was regarded as one of the pocket properties, which is thought to represent the size and shape of protein pocket.

### Protein classification

The benchmark dataset defined 5000 reverse docking cases. In each case, the top-scored protein was considered as the predicted target of the ligand. The total number of times each protein was predicted as the target of certain ligand in all the 5000 cases was calculated as the hit frequence of this protein. And if the hit probability of 100 proteins for each ligand is assumed equal, which is regarded as the ideal situation, the hit frequence of each protein should be 50 statistically (5000 divided by 100). The proteins of standard protein dataset were classified into three classes based on hit frequence: the interference proteins with hit frequence more than 50; the middle proteins with hit frequence more than 0 and less than 50; the underrated proteins with hit frequence equalling 0. The C4.5 decision tree algorithm was used to classify the interference and underrated proteins based on the 10 descriptors of protein pockets, aiming to find the simplest tree model which could use the smallest number of descriptors to predict whether the protein is overrated or underrated.

## Results and discussion

### Reverse docking score analysis of benchmark

**Fig 1:**
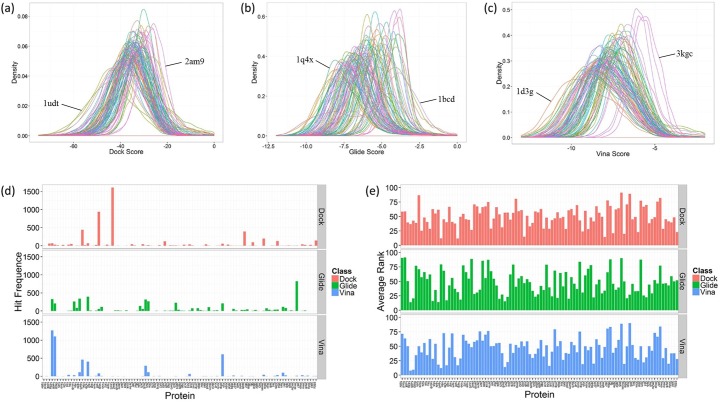
The reverse docking results of 5000 molecules. The probability density curves of (a) DOCK score, (b) Glide score, (c) Vina score of 100 proteins (different colors represent different proteins). (d) The hit frequence, (e) the average rank of 100 proteins in 5000 reverse docking cases.

**Fig 2:**
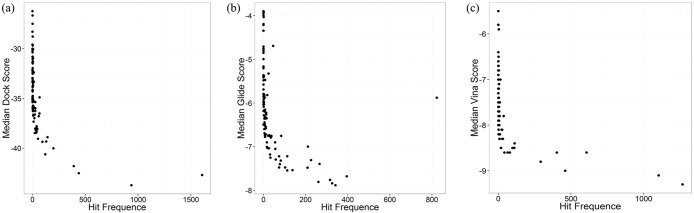
The relationship between median docking score and hit frequence of proteins. The scatter diagrams of (a) median Dock score, (b) median Glide score, (c) median Vina score of 100 proteins with their hit frequence.

The docking procedure of 5000 benchmark dataset molecules to 100 DUD-E proteins was performed by three docking programs. The docking score distribution of 100 proteins is shown in Fig 1, which demonstrates that the docking score of different proteins has a different range. In the docking by DOCK, the proteins which have the biggest difference of score are 1udt and 2am9 (PDB ID). The score range of 1udt and 2am9 is -132.5 to 278.9, -49.8 to 785.2 respectively. And their average score is -43.9 and -24.0. In the docking by Glide, the proteins which have the biggest difference of score are 1q4x and 1bcd. The score range of 1q4x and 1bcd is -11.3 to 3.2, -10.3 to 4.3 respectively. And their average score is -7.7 and -3.8. In the docking by AutoDock Vina, the proteins which have the biggest difference of score are 1d3g and 3kgc. The score range of 1d3g and 3kgc is -14.4 to 52.3, and -8.9 to -1.5 respectively. And their average score is –9.1 and -5.5. These results demonstrate that scoring functions may have different degrees of scoring bias to different proteins, some proteins resulting in a high overall docking score. In Fig 1D, we can see that some proteins show high hit frequence in 5000 reverse docking cases. Table 1 shows the proteins with top-10 hit frequence in 5000 reverse docking cases by three docking programs (The hit frequence of all proteins for three docking programs are listed in the Supporting Information S1 Table). The highest hit frequences in the reverse docking by DOCK, Glide and AutoDock Vina are 1607, 823 and 1267, while the corresponding proteins are 1zw5, 3max and 1d3g, respectively. The frequency hits are not the same in three docking procedures, which illustrates that the scoring functions of different docking programs may be sensitive to different properties of protein pockets, resulting in scoring bias to different proteins. Since the benchmark dataset molecules were chosen randomly from a vast compound library, their probability of binding with certain proteins among these 100 proteins were thought to be quite low and several actual ligands wouldn’t make a difference to the result. So they are regarded as decoys or background molecules, and the hit frequence of each protein for these molecules shouldn’t have a high value. In the ideal situation, the proteins should have an equal hit frequence of 50, assuming that they statistically have the same probability of being predicted as the target of one ligand while the activity is unclear. Thus, these proteins with such high hit frequence are probably false positives in reverse docking. Fig 1E shows that these 100 proteins have quite different average rank in 5000 reverse docking cases. The average rank of these proteins has a range of 12 to 91, 14 to 91 and 8 to 90 in the reverse docking by DOCK, Glide and AutoDock Vina, which means the overall rank of these proteins are quite different. As is shown in Fig 2, there is a positive correlationship between hit frequence and median docking score. Because average score is easily influenced by some discrete points far from average level, the median score is used here instead of average score to represent the mean level of docking score of each protein. The proteins with high hit frequence are scored higher to most ligands than those with low hit frequence, which is regarded as scoring bias in reverse docking. These frequency hits would interfere in the recognition of true targets, resulting in lots of false positives in target fishing.

**Table 1:** The proteins with top-10 hit frequence in 5000 reverse docking cases.

Dock		Glide		Vina	
Protein	Hit Frequence	Protein	Hit Frequence	Protein	Hit Frequence
1zw5	1607	3max	823	1d3g	1267
1udt	937	1s3b	395	1e66	1102
1qw6	438	1q4x	342	3chp	607
3eqh	391	1d3g	326	1qw6	460
3hmm	198	2hv5	316	1s3b	402
830c	142	2hzi	266	2hv5	291
3l3m	130	1mv9	261	2hzi	112
2oi0	121	2p2i	226	1q4x	110
3frj	94	3chp	211	3lan	97
1s3b	67	1e66	209	1udt	79

### Analysis of protein pocket properties

**Fig 3:**
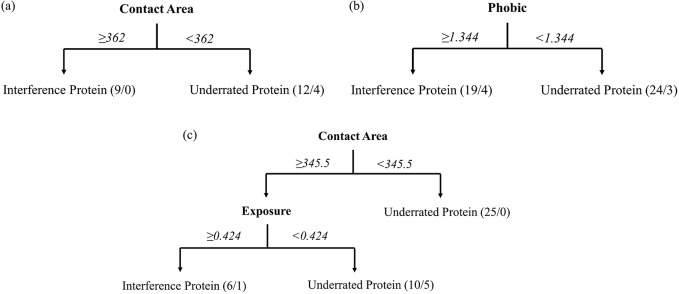
The property analysis by decision trees prediction. The decision trees analysis of interference proteins and underrated proteins in the reverse docking by (a) DOCK, (b) Glide, (c) AutoDock Vina.

To find out the protein pocket properties that cause the scoring bias to certain proteins, the proteins were devided into three classes: interference proteins, middle proteins and underrated proteins. And the properties of these three classes of protein pockets were analyzed (The properties of all protein pockets are listed in the Supporting Information S2 Table). According to the classification predictions of interference proteins and underrated proteins by decision trees (Fig 3), we found that in the reverse docking by DOCK, these two classes of proteins could be devided easily by median contact area with a high accuracy of 84.0%. And in the reverse docking by Glide, the two classes of proteins could be devided by phobic with the accuracy of 86.0%. In the reverse docking by AutoDock Vina, the two classes of proteins could be devided by median contact area and exposure with the accuracy of 87.2%. As is shown, median contact area is the root node of decision trees in the reverse docking by both DOCK and AutoDock Vina, which illustrates that median contact area plays a key role in the distinguishing of interference proteins and underrated proteins. We can see that the proteins with a large median contact area are likely to be interference proteins while those with a small median contact area are probably underrated proteins. As we know, median contact area reflects the size and shape of protein pockets. In other words, the proteins with the size and shape that can easily form large contact area with ligands are probably interference proteins in reverse docking. And in the reverse docking by AutoDock Vina, the exposure of protein pockets also helps to distinguish these two classes of proteins. If median contact area is greater than 345.5, the proteins with exposure greater than 0.424 are probably interference proteins and those with exposure smaller than 0.424 are probably underrated proteins, which means the degree of exposure involves in the producing of scoring bias. In the reverse docking by Glide, phobic (representing hydrophobicity) is the root node of decision trees, illustrating that phobic is the main property to cause scoring bias of Glide. If the proteins have pockets with high phobic, they are likely to be interference proteins in the reverse docking by Glide.

**Fig 4:**
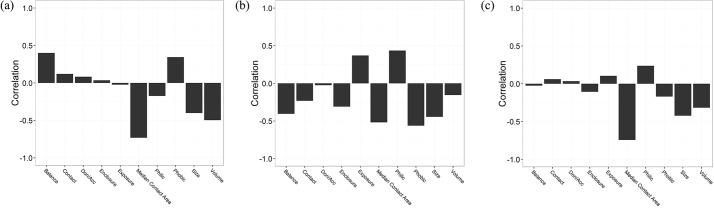
The correlationship between median docking score and the properties of protein pockets. The reverse docking by (a) DOCK; (b) Glide; (c) AutoDock Vina.

**Fig 5:**
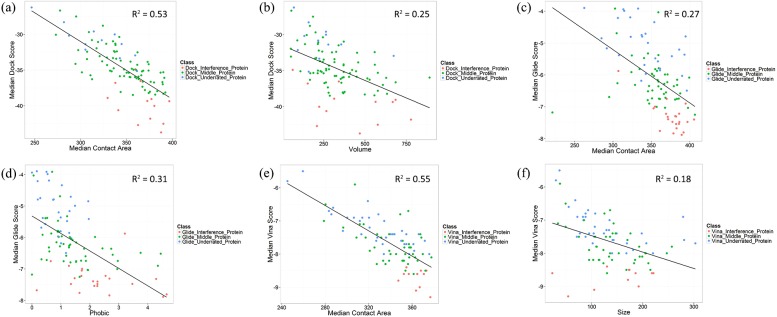
The relationship between median docking score and the highly relevant protein pocket properties of three classes of proteins. The scatter diagram of median DOCK score with (a) median contact area, (b) volume of protein pockets. The scatter diagram of median Glide score with (c) median contact area, (d) phobic of protein pockets. The scatter diagram of median Vina score with (e) median contact area, (f) size of protein pockets. The fitting lines of scatter points are shown in the diagram.

**Fig 6:**
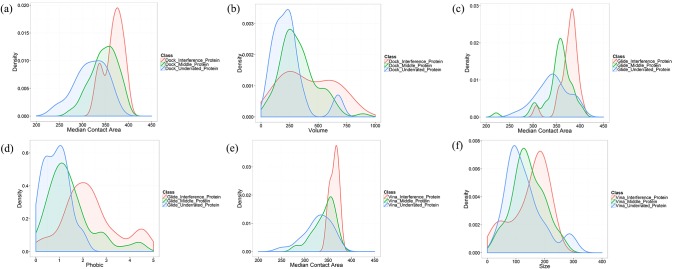
The probability density curves for the highly relevant protein pocket properties of three classes of proteins. The distribution of (a) median contact area, (b) volume of three classes of protein pockets in the reverse docking by DOCK. The distribution of (c) median contact area, (d) phobic of three classes of protein pockets in the reverse docking by Glide. The distribution of (e) median contact area, (f) size of three classes of protein pockets in the reverse docking by AutoDock Vina.

We futher analyzed the correlationship between median docking score and the properties of protein pockets. It is shown that median contact area and volume have strong negative correlationship with median docking score in the reverse docking by DOCK, which are -0.73 and -0.50, respectively (Figs 4A, 5A and 5B). And in the reverse docking by Glide, phobic and median contact area have strong negative correlationship with median docking score, which are -0.56 and -0.52 (Figs 4B, 5C and 5D). In the reverse docking by AutoDock Vina, median contact area and size have strong negative correlationship with median docking score, which are -0.74 and -0.42 (Figs 4C, 5E and 5F). Moreover, Fig 5A, 5C and 5E show that the proteins with extremely low or high docking score are almost distributed on the corresponding regions with small or large contact area, which means contact area could be the main reason causing scoring bias in reverse docking. The distribution of pocket properties which have strong correlationship with median docking score are shown in Fig 6. We can see that the median contact area distribution of interference proteins is larger than that of middle proteins and underrated proteins on the whole in the reverse docking by these three programs. Besides, the overall distribution of volume in the reverse docking by DOCK, the overall distribution of phobic in the reverse docking by Glide and the overall distribution of size in the reverse docking by AutoDock Vina also have some differences between these three classes of proteins that the interference proteins have much bigger volume, phobic and size than middle and underrated proteins on the whole. As is known, contact area has positive correlationship with volume and size. Thus, it is thought that the proteins with large pocket which can form large contact area with ligands would be likely to be scored high and become interference proteins. In addition, the scoring function of Glide also has scoring bias to the proteins with strong-hydrophobicity pocket. In conclusion, the size, shape and hydrophobicity of protein pockets are the possible properties that would cause “interprotein scoring noise” in reverse docking.

### Reverse docking score normalization

#### Score normalization strategy

We have observed the scoring bias of three docking programs in reverse docking. To eliminate this scoring bias, data normalization method was utilized to process the reverse docking score. In statistics, data normalization can transform the data with different units or dimensions to the canonical form with direct comparability. Here we used the normalization method to transform the incomparable docking score of different proteins to the normalized score that was directly comparable between proteins, and optimized the protein ranking in the reverse docking. The normalization procedure was implemented by the median docking score and the standard deviation (SD) of each protein as the following:

$$\mu_{j}=\Sigma_{i}(S_{ij})/N\;i=1,2,\ldots ,N$$ (5)

$$m_{j}={Median}_{i}\;(S_{ij})\;i=1,2,3,\ldots ,N$$ (6)

$${\left(\sigma_{j}\right)}^{2}=\Sigma_{i}{\left(S_{ij}-\mu_{j}\right)}^{2}/(N-1)\;i=1,2,\ldots ,N$$ (7)

$${S_{ij}}^{\prime }=(S_{ij}-m_{j})/\sigma_{j}$$ (8)

Where *i*, *j* is the index of each ligand and each protein; *N* is the sum of ligands; *μ*_*j*_, *m*_*j*_, and *σ*_*j*_ is the average docking score, the median docking score, and the SD value of each protein; *S*_*ij*_ and *S*_*ij*_′ is the raw docking score and the normalized docking score of each protein with each ligand.

#### Score normalizaiton results of benchmark dataset

**Fig 7:**
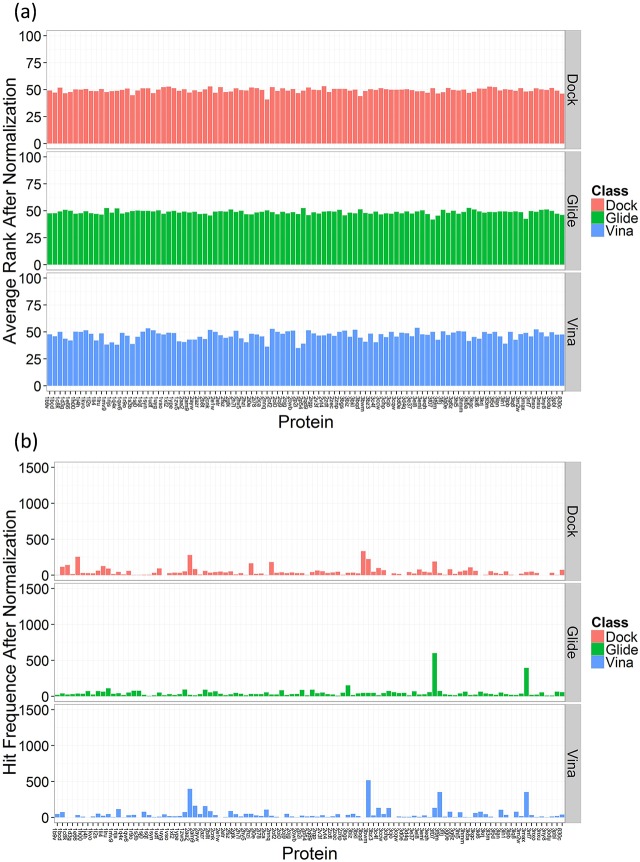
Analysis of score normalization results for benchmark dataset. (a) The average rank, (b) the hit frequence of 100 proteins in 5000 reverse docking cases.

The docking score of benchmark dataset molecules to 100 proteins were normalized to eliminate the scoring bias. Fig 7 shows the normalization results for benchmark dataset. We can see that the average rank of each protein is more balanced (Fig 7A), slightly fluctuating around the ideal value of 50 (the ranges of average rank in the reverse docking by DOCK, Glide, and AutoDock Vina are 41 to 53, 42 to 53, and 35 to 54, respectively). It is thought that the interprotein scoring noises were eliminated by the score normalization method. Thus the normalized score would be fairer to each protein and could be directly comparable. It is shown that the hit frequence of each protein is more balanced as well (Fig 7B). The SD values of hit frequences for 100 proteins were calculated to reflect the discrete degree. The SD values before score normalization for DOCK, Glide, and AutoDock Vina are 192, 114, and 193 while they are 60, 70, and 85 after score normalization, which means that the differences of the overall docking score of 100 proteins declined. And the highest hit frequences for DOCK, Glide, and AutoDock Vina are 334, 602, and 518, respectively, which are significantly lower than those before score normalization. It means that the interference degree of the interference proteins is reduced. In other words, the score normalization may help to decrease the false positives (namely interference proteins) in reverse docking.

**Fig 8:**
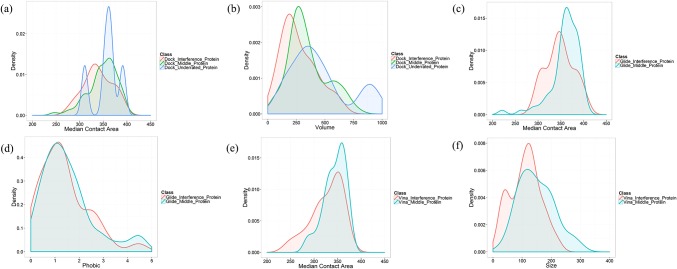
The probability density curves for the properties of three classes of protein pockets after score normalization. The distribution of (a) median contact area, (b) volume of three classes of protein pockets after score normalization for DOCK. The distribution of (c) median contact area, (d) phobic of three classes of protein pockets after score normalization for Glide. The distribution of (e) median contact area, (f) size of three classes of protein pockets after score normalization for AutoDock Vina.

After score normalization, there are still some interference proteins with relative high hit frequence. The proteins were reclassified into three classes according to the hit frequence after score normalization. And the previous high-correlationship properties were further analyzed. In Fig 8, we can see that three classes of proteins have similar distributions of properties for DOCK, Glide, and AutoDock Vina. The density curves of underrated proteins fluctuate greatly, for there are few underrated proteins for DOCK. Especially, there is none underrated proteins in the reverse docking by Glide and AutoDock Vina after score normalization. Thus, we can assume that the interprotein scoring noises caused by the properties of protein pockets were basically eliminated. Although there are still interference proteins after score normalization, it is not caused by the scoring bias to these protein pocket properties.

### Validation with Astex Diverse Set

#### Astex Diverse Set refinement

Astex Diverse Set was utilized to validate the effect of score normalization in the reverse docking by DOCK, Glide, and AutoDock Vina. There are 85 diverse protein-ligand complexes in Astex Diverse Set. The conformation searching ability and scoring function of current docking programs are not precise enough to reproduce the crystal conformation of all ligands. To focus on the scoring effects of docking programs, we only used the complexes with the root mean square deviation (RMSD) between docking and crystal conformation of ligand smaller than 2 Å to study the target prediction effect in the reverse docking by three docking programs. Finally, the refined Astex Diverse Set for DOCK includes 26 complexes, while the refined Astex Diverse Set for Glide and AutoDock Vina includes 65 and 45 complexes.

#### Target prediction effects in reverse docking

In each reverse docking case, every ligand in the refined Astex Diverse Set was docked to all the proteins in the refined Astex Diverse Set. Each ligand was taken as a probe to look for the matched target protein. The reverse docking of each ligand, namely cross docking, resulted in the scoring matrix of 26 × 26, 65 ×65 and 45 × 45. The protein with the best docking score was predicted as the target of probe molecule. If there is experimental evidence proving the prediction, the prediction of this reverse docking case is true. As is known, the Astex Diverse Set was built to ensure the greatest diversity of proteins with extremely low similarity between protein sequences. Thus, the ligands in Astex Diverse Set would be unlikely to bind with other non-cocrystallized proteins due to the similarity of protein pockets. Here we regard the cocrystallized protein as the only target of its ligand in the reverse docking. We obtained the target prediction accuracy of DOCK, Glide and AutoDock Vina, namely the percentage of truely predicted targets, which are 30.8%, 38.5% and 33.3%, respectively (Table 2). It shows that the reverse docking by current popular docking programs has a relatively low success rate.

**Table 2:** The target prediction results in the reverse docking by DOCK, Glide and AutoDock Vina.

Docking software	Correct cases with raw docking score	Accuracy with raw docking score	Correct cases with normalized docking score	Accuracy with normalized docking score
**DOCK**^a^	8	30.8%	6	23.1%
**DOCK**^b^	18	69.2%	18	69.2%
**Glide**^a^	25	38.5%	39	60.0%
**Glide**^b^	41	63.1%	55	84.6%
**Vina**^a^	15	33.3%	18	40.0%
**Vina**^b^	31	68.9%	33	73.3%

^a^ The protein which ranks first among all proteins is predicted as the target of a ligand.

^b^ The proteins which ranks top-5 among all proteins are predicted as the targets of a ligand.

**Fig 9:**
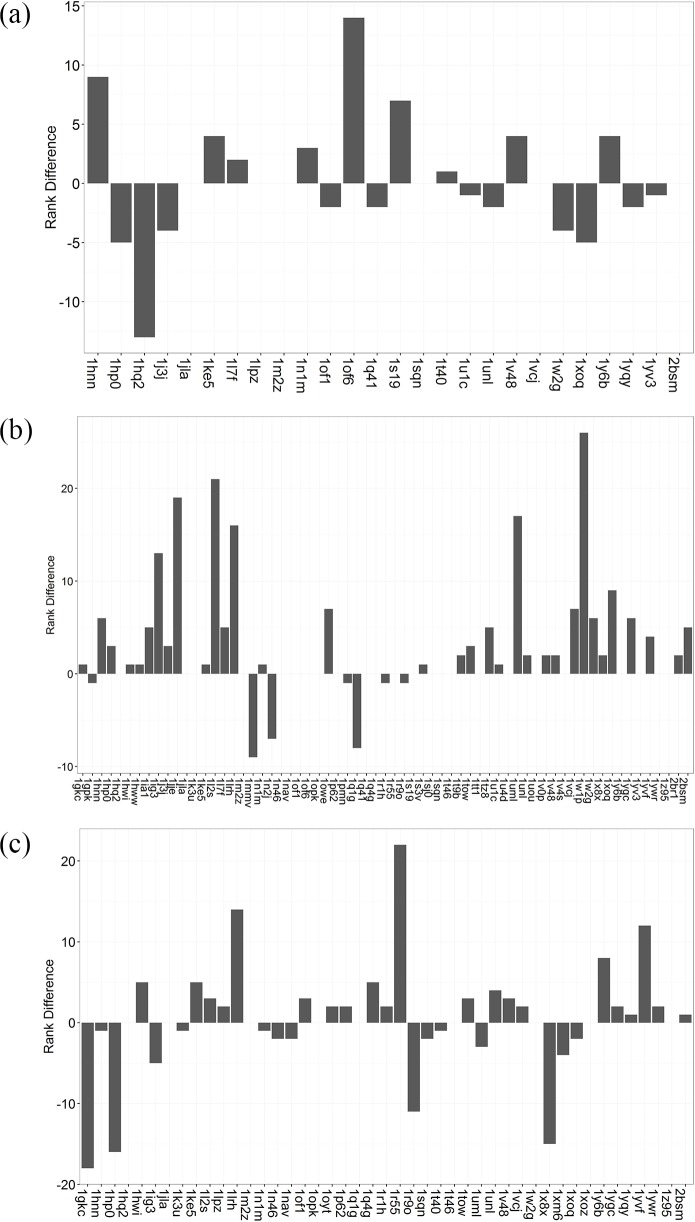
Analysis of the rank difference of cocrystallized proteins before and after the score normalization of the refined Astex Diverse Set. The rank difference of cocrystallized proteins for (a) DOCK, (b) Glide, and (c) AutoDock Vina.

The score normalization method was utilized to standardize the docking score of the refined Astex Diverse Set, aiming at eliminating the scoring bias in the reverse docking. The median docking score and the SD value of each protein with all 85 ligands were used in the score normalization. The proteins in each reverse docking case were reranked according to the normalized score. After score normalization, we retrieved the target prediction accuracy of DOCK, Glide and AutoDock Vina which are 23.1%, 60.0% and 40.0%, respectively (Table 2). The target prediction accuracy of Glide significantly increases by 21.5% (14 cases) after score normalization. To extend the restriction of being predicted as target, we regarded the top-5 scored proteins in each reverse docking case as the predicted targets. And the results of target prediction before and after the score normalization are shown in Table 2, which demonstrates the same effect of score normalization. As is seen in Table 2 and Fig 9, the ranking of most cocrystallized proteins has moved up a lot with 33 cocrystallized proteins moving up and 7 cocrystallized proteins dropping in 65 reverse docking cases for Glide (The ranking and docking score of all cocrystallized proteins before and after score normalization for three docking programs is listed in the Supporting Information S3 Table). This demonstrates that score normalization is effective to improve the target prediction accuracy in the reverse docking by Glide. However, the target prediction accuracy of DOCK decreases a little after score normalization with 9 cocrystallized proteins moving up and 11 cocrystallized proteins dropping in 26 reverse docking cases. And the target prediction accuracy of AutoDock Vina only increases slightly after score normalization with 15 cocrystallized proteins moving up and 21 cocrystallized proteins dropping in 45 reverse docking cases. Thus, the score normalization method has little effect on the improvement of target prediction accuracy of DOCK and AutoDock Vina. We suppose that DOCK and AutoDock Vina may have some difficulty in prioritizing the active ligands from inactive ligands of some proteins. Because the score normalization strategy here just aims to decrease the scoring bias to some proteins in reverse docking, it won’t be effective if the docking program couldn’t prioritize the active ligands accurately in the general docking.

#### Proteins classification analysis

**Fig 10:**
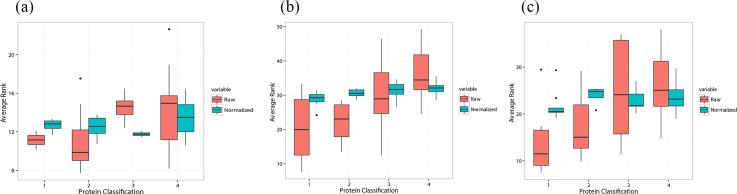
Analysis of the average ranks of four classes of proteins before and after the score normalization of the refined Astex Diverse Set. The boxplot of the average ranks for (a) DOCK, (b) Glide and (c) AutoDock Vina.

The proteins of the refined Astex Diverse Set were classified into four classes. The first class of proteins are those which have been truly predicted as the target of cocrystallized ligand and ever been falsely predicted as the target of other non-cocrystallized ligand. The second class of proteins are those which have not been truly predicted as the target of cocrystallized ligand but ever been falsely predicted as the target of other non-cocrystallized ligand. The third class of proteins are those which have been truly predicted as the target of cocrystallized ligand but never been falsely predicted as the target of other non-cocrystallized ligand. The fourth class of proteins are those which have never been predicted as the target of any ligand. The first and second classes of proteins can be regarded as the interference proteins, because these two classes of proteins would both interfere the prediction of true targets. The average rank of four classes of proteins before and after score normalization was analyzed in Fig 10. We can see that the median average rank gradually increases in these four classes of proteins before score normalization, which means that the interference proteins overall have better average rank than other proteins. Based on the previous results, it is known that the docking programs have scoring bias to these interference proteins. And after score normalization, the median average ranks of four classes of proteins are almost at the same level, which means the interference proteins are not caused by the scoring bias to specific proteins. Thus, scoring normalization could eliminate the scoring bias induced by the differences of pocket properties, resulting in the fairer docking score of each protein. Though there are still some interference proteins after scoring normalization, it might be caused by other scoring defects, such as the scoring bias to some specific protein-ligand complexes or interaction modes. In conclusion, score normalization could siginificantly increase the accuracy of the reverse docking by Glide while there is no obvious effect on DOCK and AutoDock Vina. It is supposed that DOCK and AutoDock Vina may have some difficulty in prioritizing the active ligands from inactive ligands of some proteins in general docking.

## Conclusions

In this work we carried out a large-scale reverse docking with 100 proteins and 5000 molecules using three popular docking programs: DOCK, Glide, and AutoDock Vina. From the reverse docking results, we have observed the scoring bias to specific proteins, namely interference proteins which have achieved high hit frequence. Then we analyzed the difference of pocket properties between interference proteins and the others. And we found that the interference proteins had larger contact area (related to the size and the shape of protein pockets) with ligands (for all three docking programs) or higher hydrophobicity (for Glide), which could be the possible causes of scoring bias. To eliminate the scoring bias in the reverse docking, we applied the score normalization method to transform the raw docking score into the more comparable score between different proteins. The score normalization of benchmark dataset has achieved quite obvious effect on the eliminating of scoring bias. The overall docking score of different proteins is more balanced after score normalization, which was followed by the validation of actual cases with Astex Diverse Set. The score normalization method has significantly increased the accuracy of reverse docking by 21.5% for Glide, while there were no obvious effect on the elevating of accuracy for DOCK (-7.7%) and AutoDock Vina (6.7%). We believe that these three docking programs all have scoring bias to specific proteins in reverse docking, and this scoring bias could be eliminated through score normalization. However, there might be other scoring defects in DOCK and AutoDock Vina, making it hard to prioritize the active ligands from inactive ligands in general docking of some proteins. Thus, it is not enough to only eliminate the scoring bias to specific proteins. The scoring functions of DOCK and AutoDock Vina need to be further optimized to improve the accuracy of reverse docking. Moreover, for further research the properties of protein pockets which could cause scoring bias in reverse docking could be utilized to optimize the scoring functions of docking programs, such as introducing the corresponding correction terms or adjusting the weight of different scoring terms. Therefore, there is still lots of work to improve the success rate of reverse docking for target fishing.

## Supporting information

S1 Fig The distribution of heavy atom number for ACD and different sizes of benchmark molecules. (PDF)

S1 Table The hit frequences for all proteins of standard protein dataset before and after score normalization in the reverse docking by DOCK, Glide and AutoDock Vina. (PDF)

S2 Table All protein pocket properties of standard protein dataset. (PDF)

S3 Table The ranks of all cocrystallized proteins of Astex Diverse Set before and after score normalizationin the reverse docking by DOCK, Glide and AutoDock Vina. (PDF)
