# Supplementary figures and images for: The scoring bias in reverse docking and the score normalization strategy to improve success rate of target fishing
Source: PLoS One. 2017 Feb 14;12(2):e0171433. doi: 10.1371/journal.pone.0171433 (PMC5308821; doi:10.1371/journal.pone.0171433)

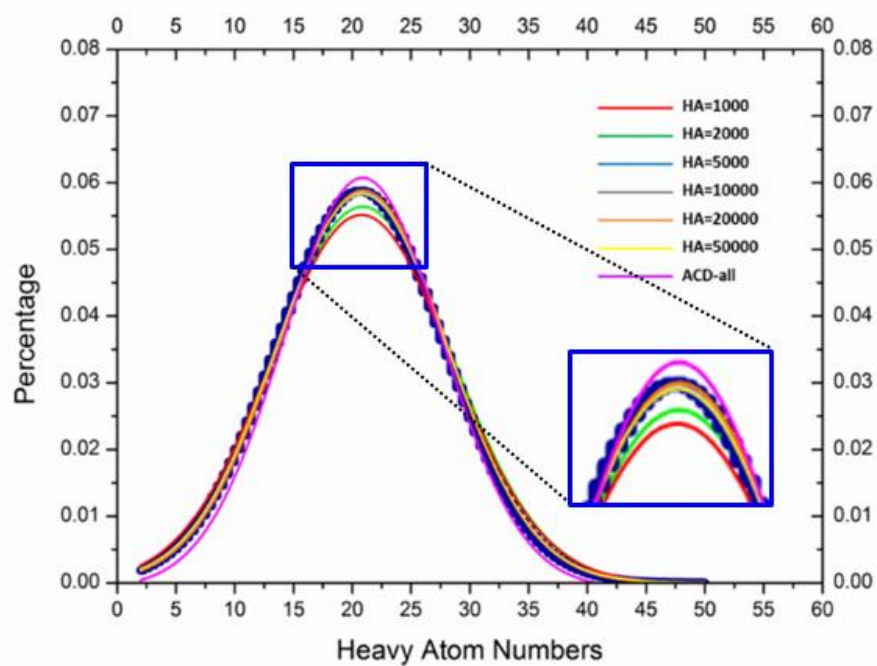

**S1 Fig. The distribution of heavy atom number for ACD and different size of benchmark molecules.**

Supplement: S1 Fig — (PDF) [file pone.0171433.s001.pdf]
