# Supplementary material for: The scoring bias in reverse docking and the score normalization strategy to improve success rate of target fishing
Source: PLoS One. 2017 Feb 14;12(2):e0171433. doi: 10.1371/journal.pone.0171433 (PMC5308821; doi:10.1371/journal.pone.0171433)
Supplement: S1 Table — (PDF) [file pone.0171433.s002.pdf]

**S1 Table. The hit frequences for all proteins of standard protein dataset before and after score normalization in the reverse docking by DOCK, Glide and AutoDock Vina.**

| Protein | Hit frequence[a] | Hit frequence[b] | Hit frequence[c] | Hit frequence[d] | Hit frequence[e] | Hit frequence[f] |
|---|---|---|---|---|---|---|
| 1b9v | 3 | 3 | 0 | 21 | 0 | 47 |
| 1bcd | 1 | 116 | 0 | 39 | 5 | 74 |
| 1c8k | 59 | 140 | 0 | 25 | 0 | 0 |
| 1d3g | 66 | 14 | 326 | 31 | 1267 | 1 |
| 1e66 | 26 | 254 | 209 | 37 | 1102 | 32 |
| 1h00 | 10 | 29 | 0 | 34 | 3 | 10 |
| 1j4h | 0 | 27 | 0 | 71 | 0 | 0 |
| 1kvo | 22 | 24 | 0 | 26 | 3 | 14 |
| 1l2s | 1 | 62 | 1 | 71 | 0 | 52 |
| 1li4 | 27 | 124 | 6 | 65 | 37 | 22 |
| 1lru | 46 | 92 | 8 | 110 | 0 | 48 |
| 1mv9 | 1 | 15 | 261 | 34 | 26 | 6 |
| 1njs | 5 | 45 | 84 | 41 | 0 | 116 |
| 1q4x | 4 | 9 | 342 | 18 | 110 | 4 |
| 1qw6 | 438 | 58 | 0 | 53 | 460 | 31 |
| 1r9o | 13 | 2 | 0 | 77 | 0 | 36 |
| 1s3b | 67 | 3 | 395 | 77 | 402 | 2 |
| 1sj0 | 0 | 6 | 11 | 19 | 4 | 80 |
| 1sqt | 4 | 4 | 1 | 6 | 0 | 33 |
| 1syn | 18 | 33 | 11 | 12 | 11 | 7 |
| 1udt | 937 | 94 | 55 | 53 | 79 | 11 |
| 1uyg | 0 | 0 | 113 | 15 | 8 | 43 |
| 1vso | 3 | 27 | 0 | 29 | 0 | 19 |
| 1xl2 | 26 | 35 | 0 | 17 | 1 | 15 |
| 1ype | 3 | 31 | 1 | 30 | 0 | 20 |
| 1zw5 | 1607 | 52 | 1 | 94 | 6 | 75 |
| 2aa2 | 1 | 279 | 16 | 19 | 1 | 399 |
| 2am9 | 2 | 83 | 16 | 14 | 3 | 162 |
| 2ayw | 1 | 7 | 5 | 29 | 0 | 48 |
| 2azr | 0 | 60 | 0 | 92 | 3 | 157 |
| 2b8t | 2 | 35 | 18 | 55 | 0 | 86 |
| 2cnk | 0 | 41 | 0 | 70 | 0 | 32 |
| 2e1w | 39 | 50 | 0 | 35 | 0 | 0 |
| 2etr | 8 | 38 | 1 | 15 | 0 | 7 |
| 2fsz | 1 | 51 | 6 | 28 | 1 | 89 |
| 2gtk | 1 | 11 | 138 | 48 | 0 | 44 |
| 2h7l | 39 | 26 | 56 | 36 | 5 | 13 |
| 2hv5 | 6 | 3 | 316 | 14 | 291 | 50 |
| 2hzi | 13 | 165 | 266 | 33 | 112 | 50 |
| 2i0e | 2 | 18 | 0 | 31 | 1 | 28 |
| 2i78 | 1 | 22 | 0 | 31 | 5 | 21 |
| 2ica | 0 | 2 | 6 | 54 | 0 | 109 |
| 2nnq | 12 | 181 | 1 | 25 | 10 | 23 |

| | | | | | | |
|---|---|---|---|---|---|---|
| 2of2 | 1 | 32 | 1 | 26 | 0 | 8 |
| 2oi0 | 121 | 39 | 9 | 85 | 0 | 0 |
| 2oj9 | 1 | 28 | 2 | 18 | 0 | 50 |
| 2ojg | 10 | 36 | 8 | 29 | 0 | 12 |
| 2owb | 8 | 27 | 8 | 32 | 1 | 4 |
| 2p2i | 7 | 28 | 226 | 89 | 6 | 25 |
| 2p54 | 2 | 3 | 33 | 12 | 0 | 12 |
| 2qd9 | 0 | 43 | 8 | 91 | 0 | 20 |
| 2rgp | 26 | 64 | 7 | 43 | 2 | 11 |
| 2v3f | 4 | 54 | 0 | 52 | 3 | 30 |
| 2vt4 | 36 | 29 | 11 | 31 | 64 | 5 |
| 2zdt | 7 | 37 | 74 | 25 | 0 | 16 |
| 2zec | 1 | 46 | 0 | 7 | 0 | 44 |
| 2znp | 1 | 5 | 78 | 50 | 0 | 0 |
| 3bgs | 1 | 31 | 24 | 152 | 0 | 35 |
| 3biz | 2 | 34 | 5 | 18 | 7 | 49 |
| 3bkl | 36 | 25 | 2 | 40 | 7 | 17 |
| 3bqd | 5 | 334 | 103 | 47 | 0 | 2 |
| 3bwm | 1 | 223 | 4 | 47 | 4 | 518 |
| 3bz3 | 1 | 47 | 13 | 47 | 0 | 29 |
| 3c4f | 2 | 98 | 22 | 15 | 9 | 134 |
| 3ccw | 6 | 68 | 0 | 45 | 0 | 47 |
| 3chp | 52 | 1 | 211 | 74 | 607 | 132 |
| 3cjo | 4 | 25 | 0 | 57 | 0 | 0 |
| 3cqw | 30 | 14 | 0 | 44 | 4 | 14 |
| 3d0e | 5 | 1 | 0 | 48 | 0 | 8 |
| 3d4q | 4 | 42 | 58 | 13 | 19 | 8 |
| 3e37 | 2 | 22 | 13 | 70 | 0 | 22 |
| 3el8 | 2 | 78 | 0 | 26 | 0 | 8 |
| 3eml | 2 | 47 | 26 | 28 | 11 | 28 |
| 3eqh | 391 | 38 | 27 | 56 | 0 | 7 |
| 3f07 | 0 | 190 | 46 | 602 | 1 | 133 |
| 3f9m | 2 | 27 | 1 | 73 | 3 | 354 |
| 3frj | 94 | 4 | 7 | 27 | 7 | 11 |
| 3g0e | 2 | 80 | 28 | 20 | 0 | 79 |
| 3g6z | 0 | 18 | 4 | 16 | 0 | 6 |
| 3hl5 | 0 | 48 | 0 | 40 | 0 | 71 |
| 3hmm | 198 | 63 | 5 | 64 | 42 | 7 |
| 3kba | 0 | 108 | 74 | 18 | 3 | 3 |
| 3kgc | 1 | 60 | 31 | 22 | 0 | 63 |
| 3kl6 | 5 | 0 | 8 | 64 | 2 | 80 |
| 3krj | 2 | 4 | 35 | 40 | 11 | 45 |
| 3l3m | 130 | 55 | 38 | 32 | 31 | 11 |
| 3l5d | 2 | 29 | 0 | 21 | 0 | 0 |
| 3lan | 3 | 14 | 113 | 52 | 97 | 106 |
| 3ln1 | 3 | 52 | 82 | 31 | 23 | 36 |
| 3lpb | 7 | 6 | 4 | 19 | 0 | 0 |

| | | | | | | |
|---|---|---|---|---|---|---|
| 3lq8 | 4 | 0 | 19 | 17 | 1 | 79 |
| 3m2w | 2 | 20 | 2 | 37 | 0 | 19 |
| 3max | 5 | 43 | 823 | 396 | 14 | 354 |
| 3nf7 | 0 | 48 | 0 | 19 | 0 | 33 |
| 3nxo | 38 | 29 | 2 | 23 | 27 | 6 |
| 3nxu | 22 | 0 | 15 | 55 | 6 | 14 |
| 3ny8 | 3 | 0 | 0 | 10 | 18 | 3 |
| 3odu | 22 | 33 | 0 | 10 | 0 | 14 |
| 3pbl | 4 | 3 | 1 | 64 | 6 | 21 |
| 830c | 142 | 73 | 0 | 58 | 8 | 41 |

[a]The hit frequence for DOCK before score normalization

[b]The hit frequence for DOCK after score normalizaiton

[c]The hit frequence for Glide before score normalization

[d]The hit frequence for Glide after score normalization

[e]The hit frequence for AutoDock Vina before score normalization

[f]The hit frequence for AutoDock Vina after score normalization
