# Supplementary material for: The scoring bias in reverse docking and the score normalization strategy to improve success rate of target fishing
Source: PLoS One. 2017 Feb 14;12(2):e0171433. doi: 10.1371/journal.pone.0171433 (PMC5308821; doi:10.1371/journal.pone.0171433)
Supplement: S2 Table — (PDF) [file pone.0171433.s003.pdf]

S2 Table. All protein pocket properties of standard protein dataset.

Protein	Median_Contact_Area_DOCK	Median_Contact_Area_Glide	Median_Contact_Area_AutoDock_Vina	Size	Volume	Exposure	Enclosure	Contact	Phobic	Philic	Balance	Don/Acc
1b9v	323.40	332.69	314.14	88.00	243.87	0.46	0.82	1.02	0.50	1.32	0.38	1.24
1bcd	320.69	313.44	311.85	34.00	51.79	0.35	0.97	1.46	0.00	2.12	0.00	0.35
1c8k	337.81	336.82	334.52	54.00	153.32	0.52	0.77	1.09	1.00	0.97	1.04	0.23
1d3g	365.24	378.74	376.78	207.00	341.63	0.34	0.89	1.16	2.67	0.57	4.68	0.81
1e66	352.57	386.47	363.58	105.00	169.10	0.33	0.98	1.29	1.35	1.31	1.03	0.37
1h00	389.05	285.00	360.71	136.00	357.06	0.41	0.85	1.11	1.94	0.75	2.59	1.12
1j4h	281.83	291.81	280.54	46.00	158.81	0.69	0.67	0.74	1.30	0.53	2.45	0.62
1kvo	356.94	361.26	339.03	123.00	287.09	0.37	0.78	1.04	1.29	1.03	1.25	1.32
1l2s	347.24	324.50	313.29	124.00	422.23	0.48	0.84	1.11	0.46	1.45	0.32	0.34
1li4	354.12	357.24	357.33	257.00	628.38	0.40	0.85	1.10	0.67	1.22	0.55	0.62
1lru	343.59	340.42	320.84	106.00	279.89	0.39	0.80	1.09	0.90	1.43	0.63	0.60
1mv9	342.10	391.99	377.75	194.00	245.59	0.22	0.97	1.22	4.65	0.40	11.63	0.32
1njs	343.64	369.97	350.64	129.00	227.07	0.20	0.91	1.24	0.65	1.38	0.47	0.70
1q4x	337.79	389.56	373.11	173.00	215.06	0.25	0.97	1.25	4.53	0.67	6.79	0.30
1qw6	390.17	342.39	366.92	192.00	577.27	0.52	0.88	1.15	1.34	1.11	1.20	0.86
1r9o	390.06	376.68	306.42	279.00	577.61	0.45	0.93	1.20	1.96	0.83	2.36	0.72
1s3b	359.62	382.78	370.79	23.00	62.77	0.72	0.58	0.76	0.16	1.14	0.14	0.97
1sj0	344.64	369.10	351.30	170.00	356.38	0.32	0.89	1.15	2.79	0.57	4.86	1.99
1sqt	312.46	323.99	300.25	72.00	167.04	0.53	0.73	1.04	0.37	1.16	0.32	1.24
1syn	389.45	368.34	352.09	185.00	608.14	0.49	0.80	0.98	0.51	1.09	0.47	0.62
1udt	387.66	394.75	364.73	221.00	468.54	0.38	0.86	1.14	1.28	0.91	1.40	0.90
1uyg	361.35	382.26	352.45	95.00	159.15	0.34	0.97	1.25	2.26	0.78	2.88	0.36
1vso	336.04	323.10	311.88	84.00	264.80	0.35	0.85	1.02	0.73	1.52	0.48	0.41
1xl2	363.19	355.14	327.82	176.00	528.22	0.38	0.78	0.96	1.21	0.79	1.53	1.07
1ype	342.67	351.12	331.52	105.00	403.71	0.51	0.81	1.15	0.92	1.16	0.80	0.88
1zw5	362.58	359.05	368.47	128.00	209.23	0.30	0.97	1.17	0.06	2.01	0.03	0.45
2aa2	295.11	356.85	356.47	136.00	185.56	0.20	0.98	1.25	2.95	0.57	5.18	0.91
2am9	324.25	347.17	349.06	125.00	166.70	0.21	0.98	1.25	4.35	0.32	13.78	2.02
2ayw	294.35	294.97	283.39	93.00	370.78	0.66	0.66	0.85	0.33	0.90	0.37	1.12

2azr	305.18	306.40	296.60	49.00	93.30	0.52	0.75	1.03	0.19	2.07	0.09	0.09
2b8t	336.39	334.66	320.31	83.00	241.47	0.48	0.85	1.13	0.89	1.34	0.66	0.45
2cnk	305.20	314.63	285.68	88.00	278.17	0.52	0.74	0.90	0.54	1.23	0.44	0.55
2e1w	383.45	393.17	373.87	176.00	376.96	0.38	0.84	1.05	0.99	0.93	1.07	1.02
2etr	347.35	350.63	327.00	143.00	449.67	0.53	0.75	0.94	1.03	0.85	1.22	1.39
2fsz	318.94	351.31	338.85	141.00	217.12	0.27	0.91	1.17	3.75	0.34	11.19	3.91
2gtk	361.66	385.99	366.02	198.00	370.44	0.36	0.85	1.05	2.15	0.67	3.22	0.54
2h7l	370.01	352.90	340.09	206.00	577.61	0.43	0.91	1.19	1.72	0.94	1.84	0.52
2hv5	338.74	361.07	347.30	171.00	315.22	0.41	0.92	1.30	1.64	1.24	1.33	0.24
2hzi	335.23	371.89	354.70	130.00	197.91	0.24	0.95	1.26	2.11	0.89	2.38	0.92
2i0e	363.09	358.52	331.62	107.00	371.13	0.44	0.82	1.05	0.46	1.28	0.36	0.81
2i78	328.80	338.06	321.05	95.00	281.60	0.62	0.72	0.90	0.20	1.15	0.18	0.90
2ica	337.11	345.62	327.93	88.00	264.11	0.28	0.89	1.16	4.45	0.28	16.14	0.21
2nnq	339.46	371.10	335.99	136.00	325.51	0.25	0.95	1.14	2.18	0.78	2.78	0.50
2of2	354.36	359.62	336.04	71.00	315.22	0.54	0.79	0.99	0.90	0.95	0.94	2.07
2oi0	341.03	359.28	332.46	117.00	266.51	0.29	0.87	1.20	1.09	1.26	0.87	1.32
2oj9	360.50	367.71	354.67	104.00	215.40	0.44	0.81	1.03	1.31	0.71	1.85	1.07
2ojg	367.33	383.61	357.24	140.00	275.43	0.37	0.85	1.13	0.85	1.22	0.69	1.05
2owb	376.28	380.88	351.03	140.00	264.11	0.31	0.84	1.07	1.52	0.88	1.72	0.69
2p2i	337.21	382.23	357.05	169.00	300.12	0.28	0.90	1.19	2.68	0.67	4.00	0.88
2p54	361.96	406.83	371.63	281.00	452.07	0.39	0.88	1.14	1.65	0.93	1.76	0.79
2qd9	342.71	359.16	343.76	95.00	223.29	0.33	0.87	1.20	2.74	0.74	3.71	0.34
2rgp	375.47	385.74	362.82	199.00	365.64	0.31	0.85	1.13	1.86	0.85	2.20	0.58
2v3f	332.03	337.24	317.20	75.00	109.42	0.24	0.98	1.18	0.29	1.58	0.18	0.37
2vt4	381.28	387.62	356.87	186.00	557.38	0.47	0.87	1.12	1.18	0.90	1.32	1.27
2zdt	366.05	369.17	361.88	181.00	325.85	0.40	0.86	1.10	1.71	1.14	1.50	0.48
2zec	301.33	307.69	287.32	55.00	187.62	0.65	0.73	0.93	0.37	1.02	0.37	1.73
2znp	374.10	405.69	370.41	282.00	559.43	0.38	0.88	1.18	2.27	0.87	2.59	0.60
3bgs	350.08	345.57	340.33	77.00	213.69	0.44	0.82	1.09	1.22	1.01	1.20	0.65
3biz	357.61	362.72	338.84	115.00	265.14	0.40	0.79	1.05	0.60	0.96	0.63	0.57
3bkl	374.15	359.21	345.21	205.00	633.18	0.37	0.90	1.09	0.53	1.54	0.34	0.35

3bqd	335.24	397.08	375.76	220.00	371.47	0.30	0.95	1.24	2.26	0.76	2.98	1.98
3bwm	297.30	355.06	315.37	143.00	220.55	0.33	0.88	1.33	0.94	1.30	0.72	0.53
3bz3	355.73	370.81	349.98	141.00	281.95	0.25	0.87	1.15	1.32	0.91	1.45	1.31
3c4f	327.89	359.87	352.83	109.00	136.86	0.28	0.95	1.23	2.97	0.91	3.28	0.43
3ccw	359.14	353.80	328.92	107.00	362.89	0.53	0.78	1.03	1.03	1.06	0.97	0.35
3chp	377.13	354.68	352.39	218.00	685.31	0.48	0.91	1.20	1.73	1.04	1.66	0.76
3cjo	306.89	340.98	307.55	83.00	250.39	0.37	0.80	1.10	0.32	1.74	0.19	0.35
3cqw	392.26	386.30	358.40	110.00	369.07	0.50	0.88	1.15	0.92	1.25	0.73	0.87
3d0e	368.24	394.63	361.16	169.00	445.90	0.38	0.88	1.15	0.75	1.21	0.62	0.84
3d4q	360.91	381.13	353.86	168.00	352.95	0.40	0.83	1.12	1.47	0.90	1.65	0.91
3e37	307.02	305.79	296.30	134.00	558.75	0.46	0.87	1.12	1.29	1.22	1.06	0.51
3el8	373.00	390.05	354.70	207.00	458.93	0.42	0.82	1.09	1.36	0.88	1.56	1.05
3eml	373.98	397.77	365.00	103.00	231.18	0.29	0.90	1.17	1.94	0.79	2.45	0.44
3eqh	375.82	383.22	352.89	303.00	778.27	0.41	0.87	1.16	1.53	1.02	1.50	0.58
3f07	287.28	304.67	280.34	49.00	132.74	0.60	0.82	1.20	0.97	1.15	0.84	0.39
3f9m	317.35	302.59	307.35	38.00	51.11	0.33	0.99	1.59	0.70	1.39	0.50	0.22
3frj	396.57	383.58	353.66	247.00	667.82	0.42	0.82	1.02	0.59	1.28	0.46	0.46
3g0e	328.69	350.88	332.85	102.00	204.77	0.49	0.73	0.93	1.70	0.74	2.31	0.73
3g6z	359.48	366.37	342.14	149.00	673.65	0.47	0.87	1.09	1.85	0.63	2.96	2.14
3hl5	246.58	262.47	245.31	30.00	69.63	0.59	0.63	0.90	0.17	1.04	0.16	0.88
3hmm	380.31	358.22	361.40	130.00	222.95	0.30	0.89	1.17	1.53	0.89	1.73	0.62
3kba	323.02	377.47	367.59	147.00	216.78	0.25	0.98	1.25	3.47	0.51	6.82	1.91
3kgc	277.56	221.00	259.49	37.00	54.19	0.36	1.00	1.59	0.00	2.63	0.00	0.18
3kl6	310.70	328.73	308.74	107.00	316.59	0.65	0.71	0.96	1.28	0.80	1.61	1.06
3krj	333.80	345.26	333.35	114.00	237.70	0.45	0.81	1.09	1.33	0.85	1.56	1.38
3l3m	372.58	361.14	335.52	211.00	563.89	0.41	0.88	1.14	1.09	1.10	1.00	0.87
3l5d	312.29	322.44	310.25	19.00	90.55	0.76	0.69	0.84	0.59	0.81	0.73	2.63
3lan	337.95	386.22	368.08	174.00	340.26	0.43	0.81	1.11	2.54	0.62	4.10	0.82
3ln1	308.96	371.31	357.33	134.00	200.65	0.31	0.95	1.23	4.29	0.60	7.18	0.83
3lpb	379.92	379.34	368.25	132.00	254.16	0.25	0.87	1.10	1.80	0.76	2.36	0.52
3lq8	366.78	367.84	358.80	193.00	371.47	0.45	0.82	1.11	1.93	0.81	2.39	0.90

3m2w	352.32	353.40	334.28	135.00	249.70	0.31	0.81	1.00	1.34	0.82	1.64	1.09
3max	273.03	306.94	278.83	85.00	147.15	0.42	0.88	1.34	3.21	0.96	3.35	0.45
3nf7	314.29	345.52	319.28	68.00	264.11	0.63	0.71	0.98	1.49	0.69	2.14	1.36
3nxo	390.17	366.85	356.20	183.00	498.72	0.40	0.84	1.12	0.97	1.08	0.90	1.07
3nxu	353.36	347.01	325.37	257.00	891.11	0.41	0.91	1.14	2.44	0.63	3.85	0.50
3ny8	391.20	396.17	372.22	215.00	514.50	0.48	0.89	1.18	1.18	0.99	1.19	0.94
3odu	367.59	361.28	333.97	168.00	685.66	0.44	0.87	1.09	0.99	1.00	0.99	1.45
3pbl	387.10	399.38	369.81	153.00	409.20	0.37	0.90	1.17	1.40	0.99	1.41	2.51
830c	329.40	342.68	327.44	123.00	313.50	0.48	0.76	1.08	0.90	0.99	0.91	1.14
