# Supplementary material for: The scoring bias in reverse docking and the score normalization strategy to improve success rate of target fishing
Source: PLoS One. 2017 Feb 14;12(2):e0171433. doi: 10.1371/journal.pone.0171433 (PMC5308821; doi:10.1371/journal.pone.0171433)
Supplement: S3 Table — (PDF) [file pone.0171433.s004.pdf]

S3 Table. The ranks of all cocrystallized proteins of Astex Diverse Set before and after score normalization in the reverse docking by DOCK, Glide and AutoDock Vina.

Docking software	Protein	Raw score	Rank before score normalization	Normalized score	Rank after score normalization
DOCK	1hnn	-43.16	11	-1.21	2
	1hp0	-39.22	3	-0.14	8
	1hq2	-34.80	3	0.68	16
	1j3j	-59.46	5	-1.91	9
	1jla	-54.47	1	-2.38	1
	1ke5	-40.62	8	-0.57	4
	1l7f	-45.34	5	-0.96	3
	1lpz	-69.99	2	-2.62	2
	1m2z	-56.44	1	-1.92	1
	1n1m	-41.96	11	-0.73	8
	1of1	-37.10	3	-0.03	5
	1of6	-34.26	16	-0.59	2
	1q41	-39.17	3	-0.06	5
	1s19	-35.67	13	-0.41	6
	1sqn	-40.77	1	-0.73	1
	1t40	-49.29	2	-2.00	1
	1u1c	-43.20	3	-0.75	4
	1unl	-49.19	1	-1.52	3
	1v48	-40.73	12	-0.72	8
	1vcj	-68.06	1	-3.02	1
	1w2g	-39.72	1	-0.15	5
	1xoq	-43.24	5	-0.27	10
	1y6b	-61.61	6	-3.26	2
	1yqy	-37.04	10	-0.01	12
	1yv3	-41.97	1	-0.54	2
	2bsm	-44.60	1	-0.84	1
Glide	1gkc	-6.93	3	-0.73	2
	1gpk	-8.48	2	-1.40	3
	1hnn	-7.67	13	-1.25	7
	1hp0	-7.25	24	-0.52	21
	1hq2	-11.81	1	-3.00	1
	1hwi	-10.20	2	-4.94	1
	1hww	-7.56	2	-1.85	1
	1ia1	-8.01	6	-1.08	1
	1ig3	-7.42	15	-1.11	2
	1j3j	-7.92	5	-0.83	2
	1jje	-7.39	20	-2.26	1
	1jla	-13.05	1	-2.52	1
	1k3u	-12.53	1	-3.20	1

1ke5	-10.42	2	-2.54	1
1l2s	-6.70	26	-0.87	5
1l7f	-6.74	9	-1.58	4
1lrh	-7.58	17	-1.68	1
1m2z	-11.94	1	-2.88	1
1mmv	-3.73	20	2.41	29
1n1m	-7.83	2	-2.13	1
1n2j	-5.37	7	0.84	14
1n46	-12.31	1	-2.64	1
1nav	-11.60	2	-2.18	2
1of1	-10.89	1	-2.81	1
1of6	-10.31	1	-2.09	1
1opk	-12.82	1	-3.21	1
1owe	-8.48	8	-2.24	1
1p62	-10.05	1	-2.89	1
1pmn	-11.00	1	-2.57	2
1q1g	-8.11	7	-0.65	15
1q41	-11.01	1	-2.78	1
1q4g	-10.96	1	-2.86	1
1r1h	-11.12	1	-3.41	2
1r55	-7.80	1	-2.31	1
1r9o	-7.89	13	-0.80	14
1s19	-13.25	1	-3.34	1
1s3v	-9.30	2	-1.76	1
1sj0	-12.33	1	-3.52	1
1sqn	-10.76	1	-2.37	1
1t46	-12.97	1	-4.48	1
1t9b	-6.93	14	-0.29	12
1tow	-8.62	4	-2.00	1
1tt1	-9.69	1	-2.33	1
1tz8	-8.73	6	-2.01	1
1u1c	-8.40	2	-1.99	1
1u4d	-10.08	1	-2.95	1
1uml	-8.49	20	-2.54	3
1unl	-9.22	4	-1.91	2
1uou	-10.13	1	-2.35	1
1v0p	-9.48	4	-2.51	2
1v48	-10.06	3	-3.01	1
1v4s	-12.16	1	-2.83	1
1vcj	-8.04	8	-2.76	1
1w1p	-5.84	50	-0.12	24
1w2g	-7.92	12	-1.48	6
1x8x	-7.52	6	-1.38	4
1xoq	-7.31	15	-0.55	6
1y6b	-10.96	1	-2.88	1

	1ygc	-7.97	7	-1.83	1
	1yv3	-10.69	1	-2.18	1
	1yvf	-8.19	5	-1.59	1
	1ywr	-11.35	2	-3.56	2
	1z95	-11.96	1	-2.71	1
	2br1	-7.68	6	-1.14	4
	2bsm	-7.97	9	-1.77	4
AutoDock Vina	1gkc	-6.60	21	1.37	39
	1hnn	-8.90	1	-0.75	2
	1hp0	-8.40	2	0.09	18
	1hq2	-9.20	1	-3.38	1
	1hwi	-9.20	6	-1.88	1
	1ig3	-7.30	7	0.42	12
	1jla	-11.60	1	-2.19	1
	1k3u	-10.00	1	-1.43	2
	1ke5	-9.40	9	-1.32	4
	1l2s	-7.80	12	-0.20	9
	1lpz	-10.30	4	-2.11	2
	1lrh	-7.90	16	-2.14	2
	1m2z	-12.40	1	-1.94	1
	1n1m	-5.80	20	1.60	21
	1n46	-11.80	1	-1.41	3
	1nav	-10.30	2	-1.05	4
	1of1	-8.50	4	-1.12	1
	1opk	-11.90	1	-2.74	1
	1oyt	-9.40	6	-1.38	4
	1p62	-7.80	3	-1.28	1
	1q1g	-9.10	1	-1.15	1
	1q4g	-9.30	6	-1.29	1
	1r1h	-9.70	3	-1.99	1
	1r55	-6.60	35	0.11	13
	1r9o	-8.70	12	-0.19	23
	1sqn	-11.60	1	-1.29	3
	1t40	-11.00	1	-1.62	2
	1t46	-13.40	1	-3.91	1
	1tow	-8.00	16	-0.20	13
	1uml	-10.30	3	-1.51	6
	1unl	-8.50	11	-0.55	7
	1v48	-8.70	4	-2.22	1
	1vcj	-7.90	3	-0.79	1
	1w2g	-9.10	1	-0.80	1
	1x8x	-6.80	14	1.32	29
	1xm6	-8.80	1	-0.27	5
	1xoq	-9.50	2	-0.67	4
	1xoz	-12.50	1	-2.48	1

1y6b	-9.40	20	-1.49	12
1ygc	-9.60	3	-2.36	1
1yqy	-9.90	2	-1.67	1
1yvf	-9.00	14	-1.42	2
1ywr	-10.60	5	-1.99	3
1z95	-10.60	3	-1.90	3
2bsm	-9.30	2	-1.84	1
